# Accuracy of global and regional longitudinal strain at peak of dobutamine stress echocardiography to detect significant coronary artery disease

**DOI:** 10.1007/s10554-020-02121-y

**Published:** 2021-01-12

**Authors:** Federica Ilardi, Ciro Santoro, Patrick Maréchal, Raluca Dulgheru, Adriana Postolache, Roberta Esposito, Giuseppe Giugliano, Anna Sannino, Marisa Avvedimento, Attilio Leone, Plinio Cirillo, Eugenio Stabile, Patrizio Lancellotti, Giovanni Esposito

**Affiliations:** 1 Division of Cardiology, Department of Advanced Biomedical Sciences, Federico II University Hospital, Via S. Pansini, 5, 80131 Napoli, NA Italy; 2 Mediterranea Cardiocentro, 80122 Napoli, Italy; 3 GIGA Cardiovascular Sciences, Department of Cardiology, University of Liège Hospital, CHU SartTilman, Liège, Belgium; 4 The Heart Hospital Baylor Plano, Plano, TX USA; 5 Gruppo Villa Maria Care and Research, Maria Cecilia Hospital, Cotignola, and Anthea Hospital, Bari, Italy

**Keywords:** Dobutamine stress echocardiography, Speckle tracking, Regional longitudinal strain, Anterior myocardial ischemia

## Abstract

Dobutamine stress echocardiography (DSE) is sensitive but subjective diagnostic tool to detect inducible ischemia. Nowadays, speckle tracking allows an objective quantification of regional wall function. We aimed to investigate the feasibility and accuracy of global (GLS) and regional longitudinal strain (RLS) during DSE to detect significant coronary stenosis (SCS). We conducted a prospective observational multicenter study including patients undergoing DSE for suspected SCS. 50 patients with positive DSE underwent coronary angiography. Besides visual regional wall motion score index (WMSI), GLS and RLS were determined at rest and at peak stress by Automated Function Imaging. DSE GLS feasibility was 96%. Among 35 patients with SCS, 12 patients were affected by multivessel disease, 18 had stenosis of left anterior descending artery (LAD), 18 of left circumflex (LCX) and 15 of right coronary artery (RCA). At peak stress, both GLS reduction (p = 0.037) and WMSI worsening (p = 0.04) showed significant agreement with coronary angiography for detecting SCS. When single lesion was considered, peak stress GLS and LAD RLS were lower in the obstructed LAD regions than in normo-perfused territories (17.4 ± 5.5 vs. 20.5 ± 4.4%, p = 0.03; 17.1 ± 7.6 vs. 21.6 ± 5.5%, p < 0.02, respectively). Furthermore, the addition of RLS to regional WMSI was able to improve accuracy in LAD SCS prediction (AUC 0.68, p = 0.037). Conversely, in presence of LCX or RCA SCS, LS was less accurate than WMSI at peak stress. In conclusion, DSE strain analysis is feasible and may improve prediction of LAD SCS, whereas regional WMSI assessment performs better in presence of SCS of LCX and RCA.

## Introduction

Dobutamine stress echocardiography (DSE) is a recognized test to detect presence and location of coronary artery disease, thanks to its good diagnostic accuracy [1, 2]. However, it remains a subjective method, limited by operators experience on image acquisition and interpretation [3–5]. Moreover, the detection of myocardial ischemia during DSE seems to be even more challenging in the presence of pre-existing wall motion abnormalities [6, 7]. In the last years, speckle tracking echocardiography has emerged as an effective tool for the quantitative assessment of regional wall function [8–11]. Accordingly, the use of this advanced technology during DSE has been proposed as a more objective method to reveal inducible ischemia, being also validated against sonomicrometry in experimental studies [12–14]. To date, few reports have described the ability of speckle tracking to detect myocardial ischemia during DSE and mostly in patients without previous regional wall motion abnormalities [15–17]. Considering the exclusion of patients with previous history of coronary artery disease from these studies, their results cannot be extended to this subset of patients. In the present study, we sought to assess the feasibility and accuracy of global (GLS) and regional longitudinal strain (RLS) during DSE to detect significant coronary artery disease in both patients with and without previous wall motion abnormalities.

## Methods

This is a prospective observational multicentre study, which included 88 consecutive patients referred for DSE to our cardiac imaging laboratory from October 2015 to December 2018. DSE was prescribed on the suspicion of obstructive coronary artery disease based on symptoms and/or the results of exercise ECG. Patients affected by acute coronary syndrome, significant valvular and congenital heart disease, atrioventricular block, persistent atrial fibrillation, complex ventricular arrhythmias, idiopathic cardiomyopathy, poor acoustic window which prevented a correct evaluation of all myocardial segments, were excluded from the study. The study protocol also included invasive coronary angiography, which was performed on an average of 12 ± 11 days following DSE. The study was conducted in accordance with the amended Declaration of Helsinki. All patients gave their written informed consent at enrollment. Patients data were collected in an anonymous way.

DSE was performed according to standardized staged protocol [18, 19] through an intravenous peripheral infusion with a mechanical pump, starting at dose of 5 µg/Kg/min and increasing at 3-min intervals to 10, 20, 30 and 40 µg/Kg/min. Intravenous atropine up to 1 mg was given at the end of the final stage if needed to increase the heart rate to the target response (85% of age-predicted maximal heart rate). ECG was monitored continuously, and blood pressure was measured at each stage. The test was considered positive in case of development of angina pectoris, new wall-motion abnormalities in at least two contiguous regional segments at any stage of dobutamine infusion, left ventricular end-systolic dilation or severe ischemic ECG changes [19].

Transthoracic echocardiography was performed with subjects in the left lateral decubitus position by experienced echocardiographers (RD, RE), using Vivid E95 and M4S transducer (General Electric Healthcare, Horten, Norway) and stored on a dedicate workstation for off-line analysis (EchoPAC, GE Healthcare). Standard 2D grey scale images of three apical views (four- and two-chamber view, and apical long-axis), parasternal long-axis and parasternal short-axis at the level of papillary muscles were acquired at rest, at low dobutamine dose (20 µg/Kg/min), at peak stress and at early recovery (within 2 min after stress). Left ventricular end-diastolic volume, end-systolic volumes and ejection fraction were measured at rest and at peak stress using Simpson’s biplane method of discs [20].

**Fig. 1:**
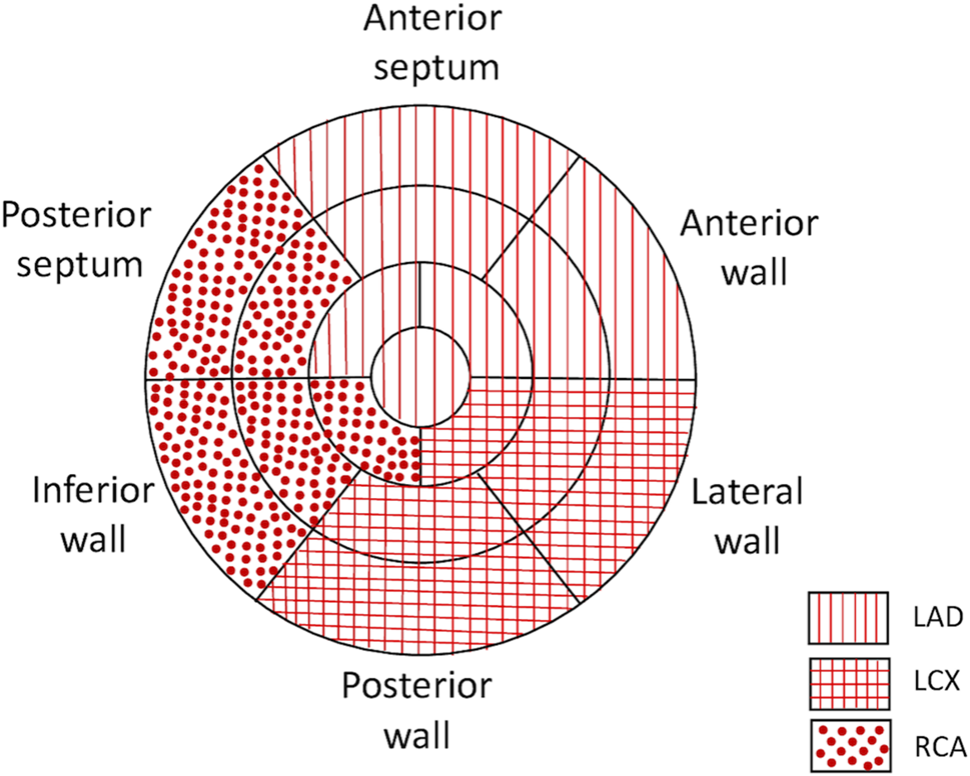
Circumferential polar plot of the 17 myocardial segments derived by visual wall motion assessment and speckle tracking echocardiography, showing the definition of regions supplied by respective coronary arteries. *LAD* left anterior descending artery; *LCX* left circumflex coronary artery; *RCA* right coronary artery

Wall motion was assessed by at least 2 experienced independent observers (FI, CS) using a 17 myocardial segment model, as recommended by guidelines [20]. A semiquantitative scoring system was used to analyse each segment (1 = normal or hyperkinetic; 2 = hypokinetic; 3 = akinetic; 4 = dyskinetic). Global wall-motion score index (WMSI) was calculated as the average of the scores assessed for each segment at rest and at each DSE stage. Figure 1 shows the 17 segments model divided according to perfusion territories of the three major coronary arteries, based on a standardized perfusion model [21]. According to this model, basal, midventricular and apical segments of anteroseptal and anterior walls, and the apex were attributed to left anterior descending artery (LAD) territory. Basal and midventricular segments of posterior wall, and all lateral wall segments (basal, midventricular and apical) were attributed to left circumflex (LCX) coronary artery territory. All inferior wall segments and basal and midventricular segments of posterior septum were attributed to right coronary artery (RCA) territory (Fig. 1). Regional WMSI for each coronary territory was calculated.

Strain analysis was based on speckle-tracking approach and measured by an experienced cardiologist blinded to the clinical history and coronary angiography results (FI, CS). In order to enable GLS analysis, two-dimensional grayscale images from the apical four-chamber, two-chamber, and long-axis views were acquired at frame rates between 50 and 80 frames/s (mean 63.1 ± 4.4 frames/s), in loops of three successive cardiac cycles. Speckle tracking was performed using the Automated Function Imaging (AFI) algorithm, that is incorporated in a quad-screen of the echo machine, and automatically analyses myocardial motion by tracking frame-to-frame speckle changes. When necessary, manual adjustments were performed to ensure correct ‘anchorage’ to the mitral annulus, to exclude papillary muscles and chordae from tracking, and to correctly include the left ventricular apex. The width of the region of interest was eventually adjusted to cover the entire myocardial wall thickness. The left ventricle was divided into six myocardial segments in each view, and GLS was calculated as the average of peak longitudinal strain of all segments at end systole [22]. RLS resulted from the sum of the territorial segmental strain divided by the number of segments visualized, applying the same perfusion model used for the regional WMSI analysis (Fig. 1). Inadequate tracked segments were automatically excluded from the analysis. When more than 2 segmental strain within the same coronary territory was not measurable, the strain analysis was considered unfeasible.

Selective coronary angiography was performed in multiple projection according to the Judkins technique, in all patients who underwent positive DSE for inducible ischemia, within 8 weeks of echocardiographic examination. All images were interpreted by experienced operators (GE, ES, PC), who based the estimation of the degree of coronary artery narrowing on visual assessment of angiograms. Significant coronary stenosis (SCS) were defined as ≥ 50% for left main coronary artery, ≥ 70% for the others epicardial arteries. When visual assessment was suggestive of intermediate coronary lesions (40–70% obstruction), fractional flow reserve was performed and SCS was defined with fractional flow reserve < 0.80.

Statistical analyses were performed using IBM-SPSS, version 23 (SPSS Inc, Chicago, IL, USA).

Continuous variables were expressed as mean ± SD and compared with unpaired Student t-test. Categorical data were expressed as percentage and comparisons were made by χ^2^ test. A p value < 0.05 was considered statistically significant. Patients were divided in two groups according to the presence of obstructive coronary artery disease detected at coronary angiography. Inter-rater agreement Kappa (κ) was used to evaluate agreement between DSE or RLS analysis and coronary angiography considered as gold standard. When there is perfect agreement, κ is 1. Receiver operating characteristic (ROC) curves were used to evaluate the diagnostic performance of RLS, regional WMSI analysis and their combination.

## Results

A total of 88 patients performing DSE were screened for the study. 33 patients were excluded from further analysis because of absence of inducible ischemia at DSE; 3 patients refused the angiographic examination; 2 patients were rejected due to suboptimal RLS quality. The final study population consisted of 50 patients with positive DSE for inducible ischemia. In 8 cases, stress test was interrupted before reaching the target heart rate because of development of angina pectoris (n = 4), occurrence of complex ventricular ectopy (n = 2) or symptomatic hypotension (n = 2) associated with new/worsening wall-motion abnormalities. All patients underwent elective coronary angiography; in 35 (70%) of them SCS was detected. During coronary angiography, the estimation of coronary stenosis severity required fractional flow reserve evaluation in 10 cases. Table 1 reports demographic, clinical and angiographic results of the study population according to the presence or absence of SCS at coronary angiography. Previous percutaneous coronary interventions or coronary artery bypass grafting were more prevalent in patients with SCS (77.1 vs. 33.3% respectively, p = 0.003).

**Table 1:** Baseline demographic data, clinical and angiographic characteristics according to the presence or absence of significant coronary artery disease

Variables	Total (n = 50)	CAD (n = 35)	No CAD (n = 15)	*P* value
Clinical variables				
Age, years	66.3 ± 8.2	67.2 ± 6.7	63.9 ± 10.7	0.190
Male, gender	41 (82%)	30 (85.7%)	11 (73.3%)	0.296
Body surface area, m^2^	1.8 ± 0.2	1.8 ± 0.2	1.8 ± 0.2	0.800
Previous CABG/PCI	32 (64%)	27 (77.1%)	5 (33.3%)	**0.003**
Risk factors				
Hypertension	38 (76%)	25 (71.4%)	13 (86.7%)	0.248
Dyslipidemia	33 (66%)	23 (65.7%)	10 (66.7%)	0.948
Diabetes	18 (36%)	12 (36.4%)	6 (40%)	0.809
Current/previous smoker	19 (38%)	15 (48.4%)	4 (30.8%)	0.282
Family history of CAD	10 (20%)	6 (19.4%)	4 (30.8%)	0.410
Chronic kidney disease	10 (20%)	7 (22.6%)	3 (21.4%)	0.931
Systolic arterial pressure, mmHg				
Rest	137 ± 17	137 ± 17	137 ± 16	0.906
Stress	142 ± 30	143 ± 32	139 ± 25	0.656
Diastolic arterial pressure, mmHg				
Rest	77 ± 8	74 ± 10	75 ± 8	0.665
Stress	69 ± 13	70 ± 13	67 ± 14	0.460
Heart Rate, bpm				
Rest	68 ± 13	68 ± 13	68 ± 14	0.892
Stress	130 ± 14	130 ± 15	130 ± 13	0.997
Cardiovascular medications				
Aspirin	38 (76%)	28 (84.8%)	10 (66.7%)	0.15
P2Y12 inhibitors	18 (36%)	14 (42.4%)	4 (26.7%)	0.29
Beta-blocker	33 (66%)	23 (69.7%)	10 (66.7%)	0.83
Statin	40 (80%)	28 (84.8%)	12 (80%)	0.68
ACEi/ARB	37 (74%)	25 (75.8%)	12 (85.7%)	0.45
Calcium Channel Blocker	12 (24%)	9 (27.3%)	3 (20%)	0.59
Nitrate	1 (2%)	1 (2.9%)	0	0.51
Single-vessels disease		23 (65.7%)		
LAD		7 (22.6%)		
LCX		9 (25.7%)		
RCA		7 (22.6%)		
Two-vessels disease		9 (25.7%)		
LAD and LCX		3 (8.6%)		
LCX and RCA		2 (5.7%)		
LAD and RCA		4 (11.4%)		
Three-vessels disease		4 (11.4%)		

Values in bold indicate statistically significant results

Data are expressed as n (%) or mean ± SD

*ACEi* angiotensin converting enzyme inhibitors; *ARB* angiotensin receptor blocker; *CABG* Coronary artery bypass grafting; *CAD* coronary artery disease; *LAD* left anterior descending artery; *LCX* Left circumflex coronary artery; *PCI* percutaneous coronary intervention; *RCA* right coronary artery

The feasibility of DSE GLS was 96% (n = 50/52). Of the 850 analyzed myocardial segments, 13 (1.5%) at peak stress were rejected due to poor tracking. Table 2 summarizes the main echo parameters at rest and during DSE. Of note, 31 (62%) patients had already wall motion abnormalities at rest, with no significant differences between the groups of patients with or without coronary artery disease (69% vs. 47% respectively, p = 0.144). Left ventricular ejection fraction and volumes, WMSI and GLS did not differ between the two groups at rest and at peak stress. Table 3 reports the concordance in detecting SCS between coronary angiography and both global and regional WMSI and LS at peak stress. The agreement at DSE peak was mild between both WMSI and GLS with coronary angiography (p = 0.041 and p = 0.037, respectively). The agreement between DSE and coronary angiography was higher for RLS for LAD SCS (p = 0.022) compared to regional WMSI (p = 0.031). More precisely, in 94.3% of cases RLS correctly identified significant LAD stenosis, compared to only 56% properly diagnosed by regional WMSI.

**Table 2:** Echocardiographic parameters according to the presence or absence of significant coronary artery disease

Variables	Total (n = 50)	CAD (n = 35)	No CAD (n = 15)	*P* value
LV EF, %				
Rest	56 ± 9	55 ± 9	59 ± 7	0.14
Stress	57 ± 10	56 ± 11	59 ± 7	0.25
End Diastolic Volume, ml				
Rest	97 ± 29	97 ± 31	96 ± 25	0.89
Stress	71 ± 26	74 ± 28	63 ± 19	0.19
End Systolic Volume, ml				
Rest	45 ± 23	46 ± 25	40 ± 16	0.36
Stress	31 ± 18	34 ± 20	26 ± 9	0.16
WMSI				
Rest	1.24 ± 0.33	1.27 ± 0.36	1.17 ± 0.25	0.30
Stress	1.47 ± 33	1.53 ± 0.35	1.34 ± 0.27	0.07
GLS, %				
Rest	19.3 ± 4.5	19.0 ± 4.7	20.2 ± 3.9	0.39
Stress	19.4 ± 5.0	18.8 ± 5.2	20.6 ± 4.6	0.26

Data are expressed as mean ± SD

*CAD* coronary artery disease; *GLS* global longitudinal strain; *LV EF* left ventricular ejection fraction; *WMSI* wall motion score index

**Table 3:** Agreement between coronary angiography and echocardiographic parameters at peak of DSE in diagnosis of significant coronary artery disease

		Coronary angiography		Agreement	
		No significant stenosis N ( %)	Significant stenosis N (%)	Kappa	P value
Global					
WMSI	Normal	3 (20%)	1 (2.9%)	0.217	**0.041**
	Diseased	12 (80%)	34 (97.1%)		
GLS	Normal	4 (26.7%)	2 (5.7%)	0.253	**0.037**
	Diseased	11 (73.3%)	33 (94.3%)		
LAD					
Regional WMSI	Normal	24 (75%)	8 (44.4%)	0.306	**0.031**
	Diseased	8 (25%)	10 (55.6%)		
RLS	Normal	4 (26.7%)	2 (5.7%)	0.322	**0.022**
	Diseased	11 (73.3%)	33 (94.3%)		
LCX					
Regional WMSI	Normal	18 (56.2%)	5 (27.7%)	0.257	**0.05**
	Diseased	14 (43.8%)	13 (72.3%)		
RLS	Normal	14 (43.7%)	7 (38.8%)	0.043	0.74
	Diseased	18 (56.2%)	11 (61.2%)		
RCA					
Regional WMSI	Normal	22 (62.8%)	3 (2.9%)	0.360	**0.005**
	Diseased	13 (37.2%)	12 (97.1%)		
RLS	Normal	20 (57.1%)	8 (53.3%)	0.034	0.80
	Diseased	15 (42.9%)	7 (46.7%)		

Values in bold indicate statistically significant results

*GLS* global longitudinal strain; *LAD* left anterior descending artery; *LCX* left circumflex coronary artery; *RCA* right coronary artery; *RLS* regional longitudinal strain; *WMSI* wall motion score index

**Fig. 2:**
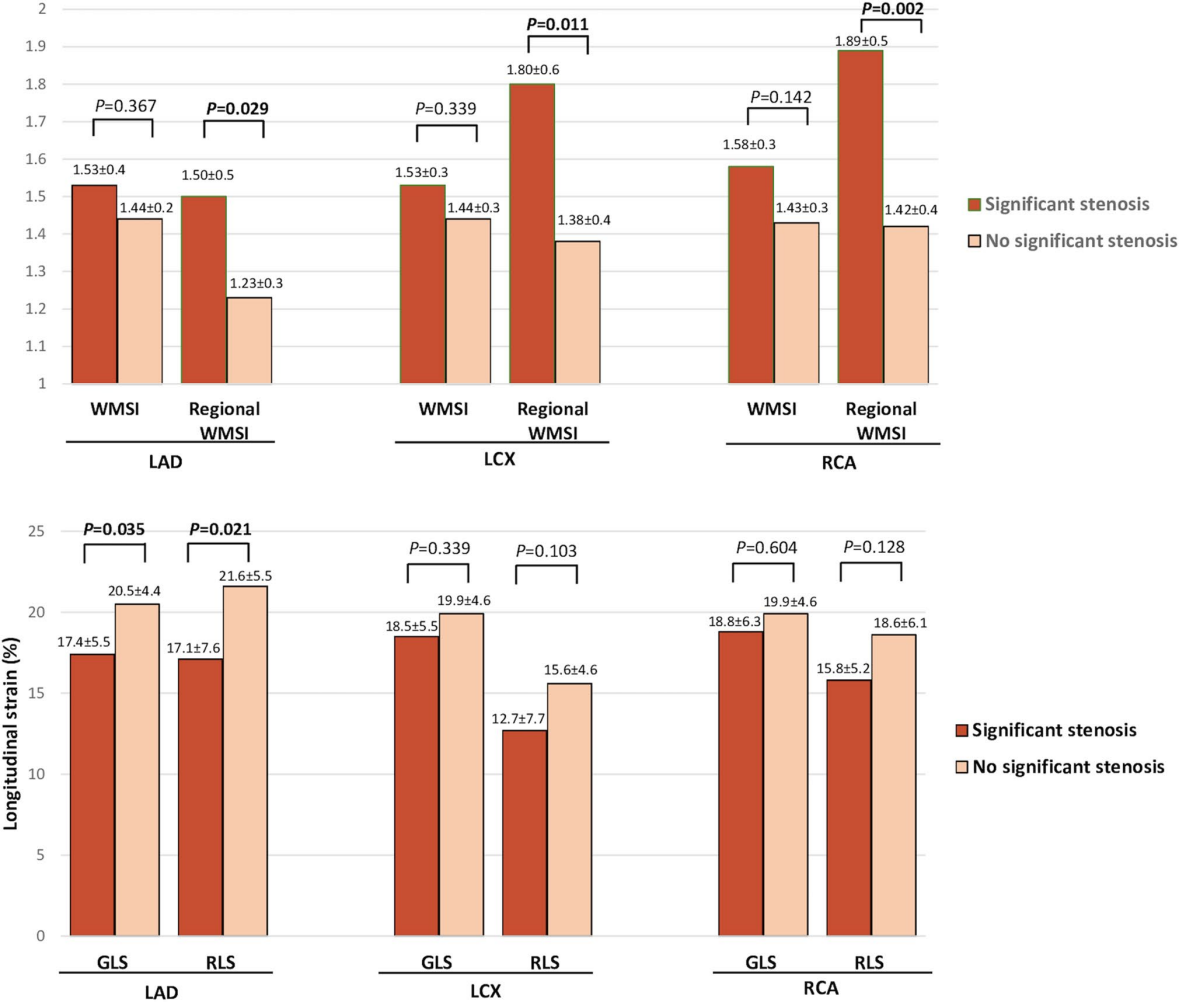
Bar graph showing comparison between global and regional WMSI, GLS and RLS according to the perfusion territory supplied by the given SCS at peak stress

When considering SCS of LCX and RCA territories, a significant, despite mild, agreement with coronary angiography was observed only with regional WMSI (p = 0.05 and p = 0.005, respectively). Figure 2 depicts comparisons results of global and regional WMSI, GLS and RLS according to the perfusion territory supplied by the given SCS at peak stress. Regional WMSI appeared to be significantly higher when SCS occurred, independently from which coronary was affected (LAD p = 0.029, LCX p = 0.011, RCA p = < 0.02). GLS and RLS were lower in the myocardial regions supplied by obstructed LAD than in regions supplied by patent LAD (GLS p = 0.035; RLS p = 0.021). Conversely, no differences were shown in strain analysis when obstruction of the LCX and RCA was observed.

**Fig. 3:**
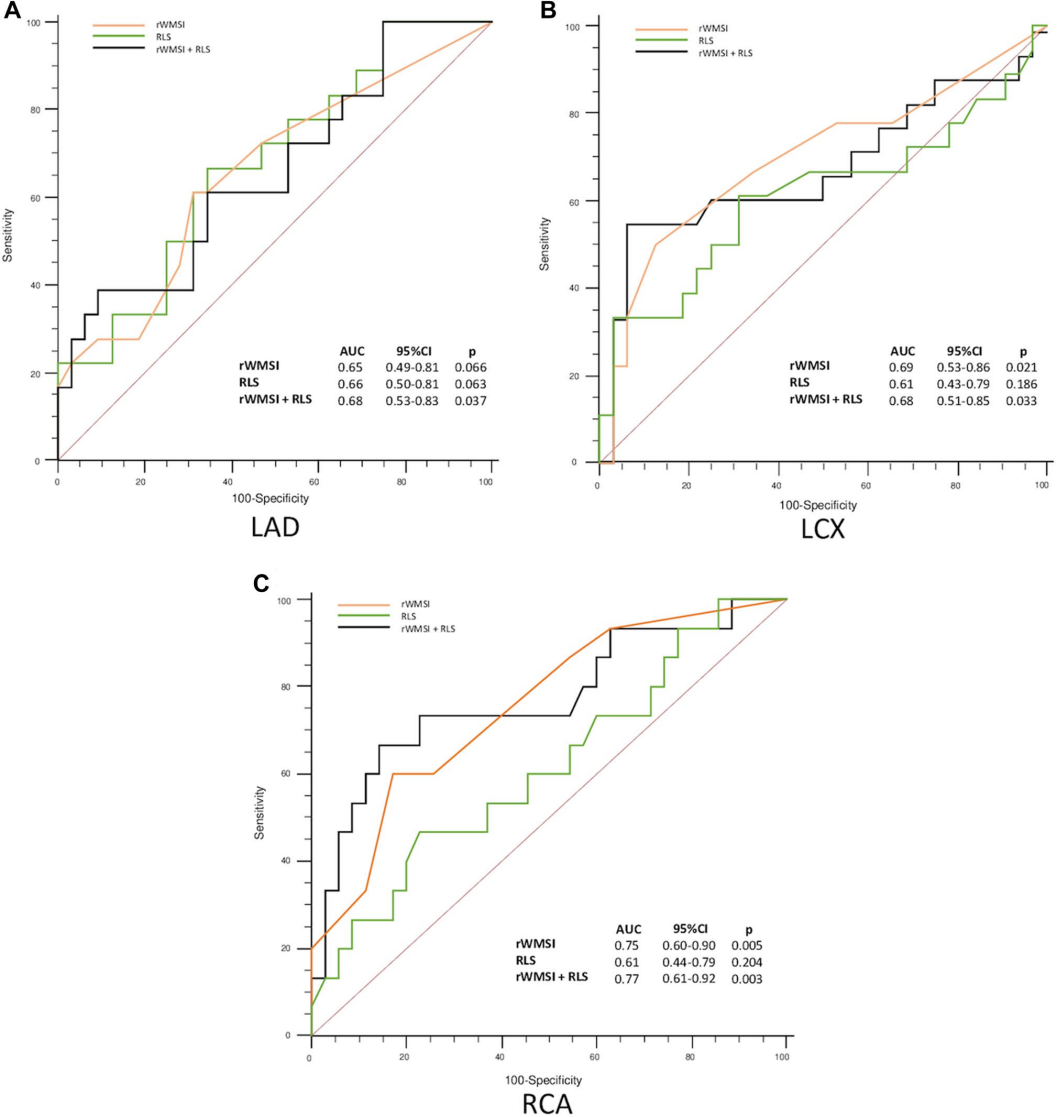
Diagnostic performance of RLS, regional WMSI and their combination for the detection of regional myocardial ischemia in territories supplied by obstructed LAD (**a**), LCX (**b**) and RCA (**c**). *AUC* area under the curve; *CI* confidence intervals; *LAD* left anterior descending artery; *LCX* left circumflex coronary artery; *RCA* right coronary artery; *rWMSI* regional wall motion score index

The ROC curves and areas under the curve (AUC) values for RLS, regional WMSI and their combination for the detection of SCS according to the perfusion territory of the three major coronary arteries are shown in Fig. 3. In territories supplied by obstructed LAD, only the combination of RLS at peak stress with regional WMSI abnormality is able to provide a statistically significant AUC value (p = 0.037, AUC = 0.68) (Fig. 3a). As expected, regional WMSI showed good accuracy in detecting ischemia in presence of LCX SCS (p = 0.021, AUC = 0.69), while the addiction of RLS to visual wall motion assessment wasn’t able to improve accuracy (p = 0.033, AUC = 0.68) (Fig. 3b). In myocardial segments supplied by obstructed RCA, instead, the addition of RLS to regional WMSI provided slightly higher accuracy in detecting SCS (p = 0.003, AUC = 0.77) compared to regional WMSI alone (p = 0.005, AUC = 0.75). (Fig. 3c).

**Fig. 4:**
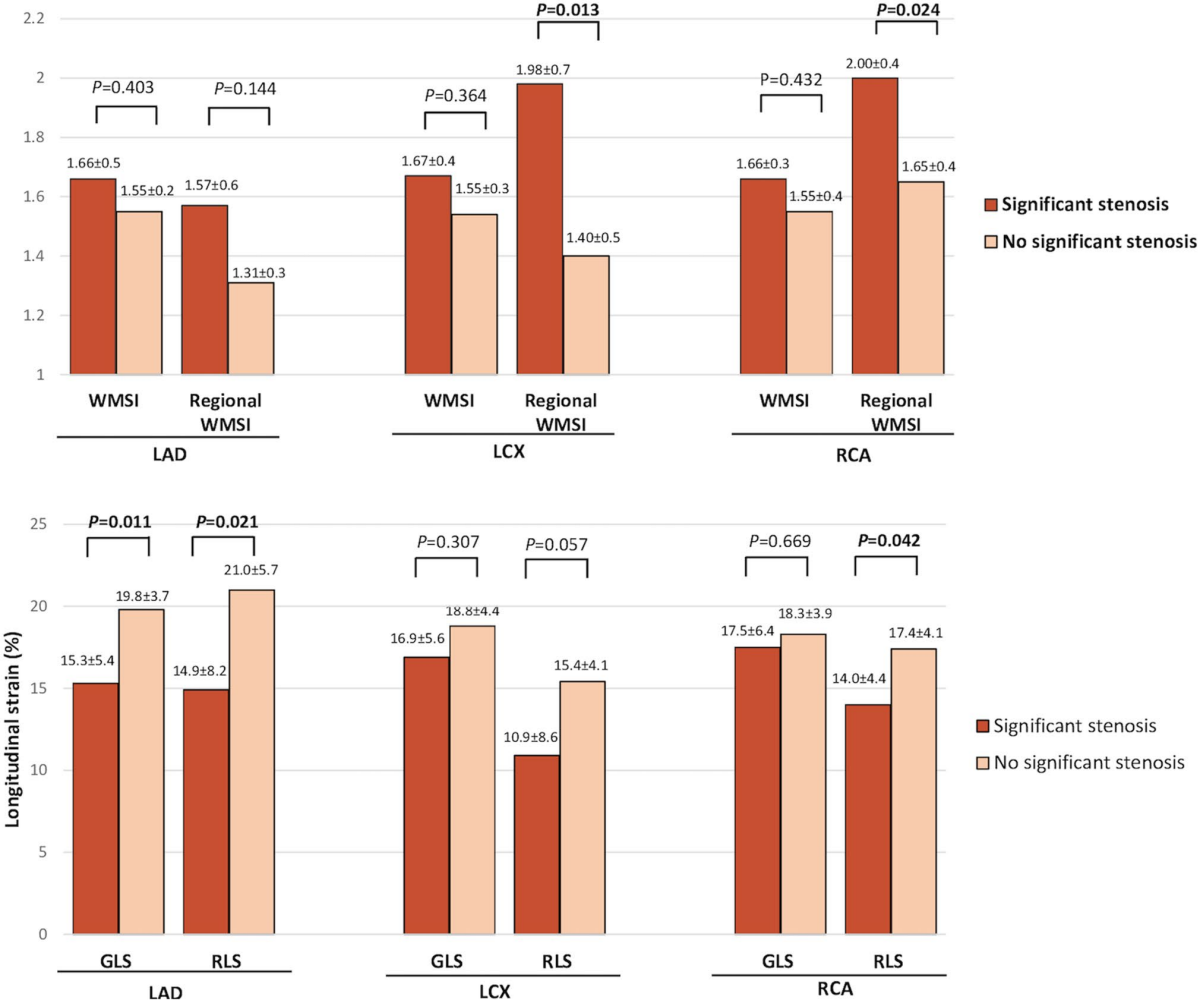
Bar graph showing comparison between global and regional WMSI, GLS and RLS according to the perfusion territory supplied by the given SCS at peak stress in patients with previous wall motion abnormalities

Interestingly, considering only those patients with resting wall motion abnormalities (n = 31), GLS and RLS – but not global or regional WMSI—were able to identify SCS of LAD coronary artery (p = 0.011 and p = 0.021, respectively) (Fig. 4). Also in this subgroup of patients, GLS and RLS could not distinguish LCX SCS. In myocardial regions supplied by obstructed RCA, instead, RLS—but not GLS—was significantly lower than in regions supplied by patent coronary artery (p = 0.042) (Fig. 4).

## Discussion

This study demonstrated that: (i) the assessment of longitudinal strain during DSE is feasible even at the elevated heart rate reached during DSE peak; (ii) GLS and RLS at DSE peak are more sensitive than visual WMSI assessment to detect inducible ischemia in the myocardial territories supplied by LAD; (iii) these results are more evident when patients with resting wall motion abnormalities are considered; (iv) in territories supplied by LCX and RCA, GLS and RLS are less reliable to detect obstructive lesions.

**Fig. 5:**
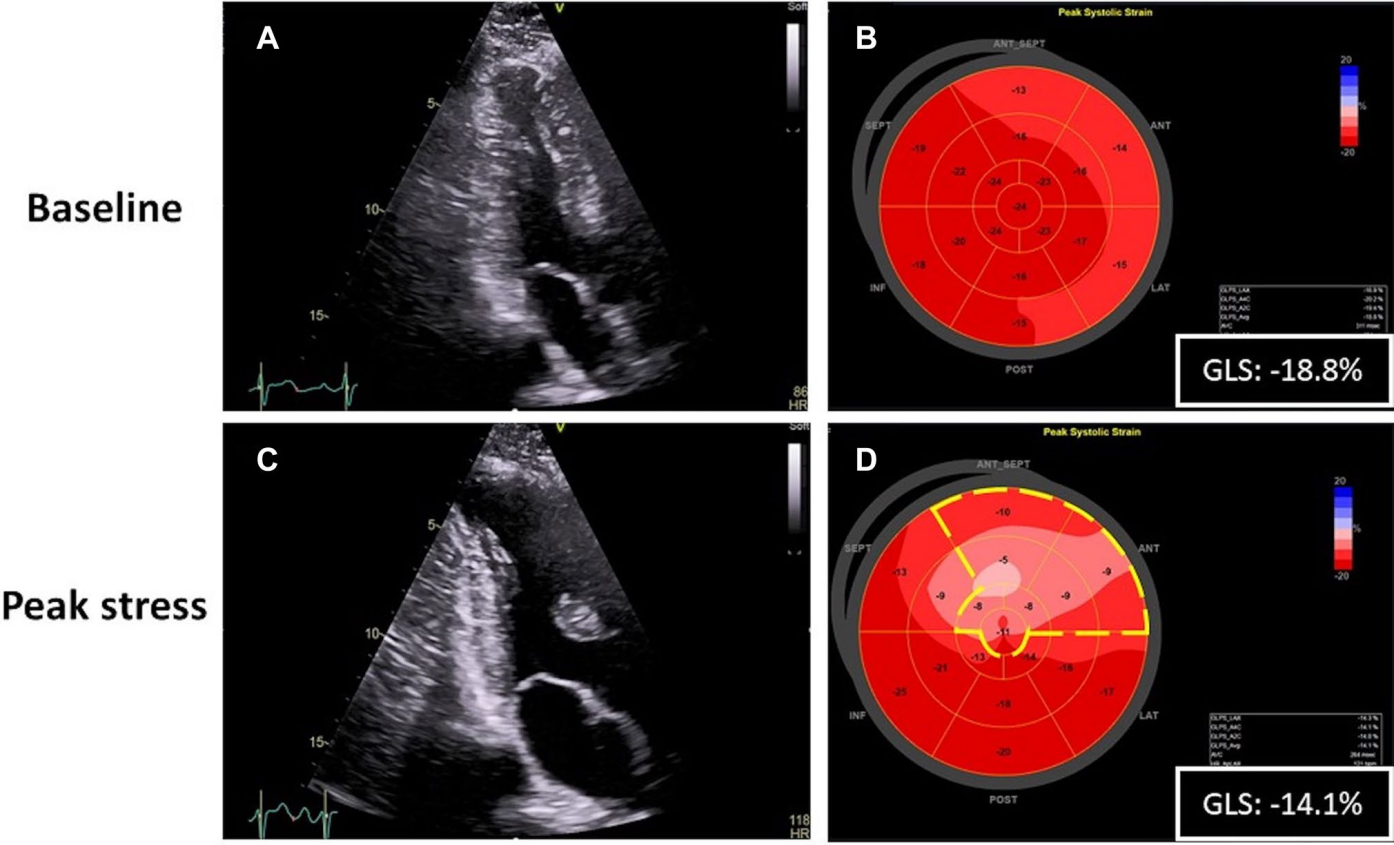
Application of AFI analysis during dobutamine stress echo in patients with severe obstruction of the LAD. Upper panels showing resting apical long-axis view during end-systole (**a**), and strain analysis performed at the same time (**b**). Lower panels peak stress apical long-axis view depicting an abnormal response with increased left ventricular end-systolic diameter and hypokinetic contraction of the mid-apical segment of the anterior portion of the septum (**c**). Strain analysis performed at peak stress showing a clear concomitant RLS reduction in the LAD territory (dotted yellow lines) and GLS reduction compared to baseline values (**d**)

Longitudinal strain analysis during DSE has been recently proposed as a quantitative method to overcome the limitation of the visual evaluation of regional wall motion. Despite an average accuracy of > 80% for detection of coronary artery stenosis [2], traditional stress echocardiography is characterized by a subjective interpretation of wall motion abnormalities, even among expert readers [1–3]. This limitation is still more evident in patients with history of coronary artery disease and previously known wall motion abnormalities, a setting in which the identification of residual and/or new areas of ischemia becomes very challenging [6, 7]. In fact, the growth of collateral circulation or the imperfect assignment of myocardial regions to coronary arteries may contribute to the underestimation of ischemia under these circumstances. In our previous experience, we have shown that AFI-derived GLS measured at DSE peak was more accurate than visual WMSI to detect inducible ischemia in patients with three vessel and left main disease [23]. The present study demonstrates that AFI technique can be performed during DSE, allowing a correct and almost complete analysis of myocardial deformation at every stages of stress protocol. AFI applied to DSE showed a feasibility of 96%, which is in line with previous literature, where feasibility ranged from 77 to 100% [19]. Non-angle dependent and better signal-to-noise ratio represent two main advantages for the use of strain analysis in the quantitative assessment of myocardial region supplied by obstructed coronary artery. Furthermore, the utilization of phased array probes and software advancement provide an enhanced visualization of structures at the sides of the sector in combination with higher frame rate. This allows to overcome the previously observed limitation of a poor speckles’ identification occurring at DSE peak [23, 24]. Moreover, the automatic localization of endocardial borders enables a faster and objective quantitative analysis of myocardial longitudinal motion. The clear result presentation with a LV bullseye displaying segmental peak strain values and a corresponding polar map represents a handy tool to identify motion abnormalities even for less experienced operators (Fig. 5).

As a result of these technical improvements, in the present study, GLS at DSE peak stress showed a moderate diagnostic accuracy in detecting SCS, as compared with the results of coronary angiography. Interestingly, when the analysis was restricted to patients with significant LAD stenosis, GLS and RLS showed a better agreement compared to WMSI in detecting SCS. Moreover, the addiction of RLS to the visual wall motion assessment of myocardial ischemia has shown to significantly improve diagnostic accuracy in predicting LAD SCS, over visual wall motion alone. This incremental value of strain imaging at DSE peak could represent an additional tool in reducing false negative results obtained by visual assessment, especially in patients with suspected LAD disease. LAD is usually the largest of the 3 epicardial coronary artery and subtends about 50% of the LV myocardial mass [25]. The presence of significant LAD disease has been associated with worse prognosis than SCS involving other coronary arteries [26, 27]. Thus, given the extent and functional relevance of the myocardial territories supplied by LAD coronary artery, a properly and timely detection of LAD stenosis represents an appealing task. To notice, the incremental value of GLS and RLS in the detection of LAD SCS seems to be even more useful in patients with previous wall motion abnormalities. In this subset of patients, indeed, strain analysis appears to be more sensitive than visual WMSI in identifying ischemia areas in myocardial segments supplied by LAD coronary artery.

The diagnostic power of both GLS and RLS was not accurate in detecting SCS of LCX and RCA territories where the visual analysis of regional wall motion showed a better agreement with coronary angiography in detecting SCS. It is commonly recognized that the ability to precisely identify a LAD obstruction during stress echocardiography exceeded that for the posterior circulation [19, 28]. Moreover, a discrepancy of the sensitivity between strain measurements in inferior and postero-lateral circulation than anterior coronary circulation had been described in previous studies [16, 17, 29]. In a study of 155 patients, Hanekom et al. reported the best sensitivity of 2D strain in LAD territories (sensitivity 77%, specificity 79%, accuracy 78%) [29]. Similarly, Aggeli et al. described a 2D-strain superior diagnostic efficiency for the evaluation of anterior coronary circulation, as compared to posterior circulation [17]. Roushdy et al. also demonstrated a superiority of 2D strain at DSE peak in detecting LAD and RCA lesions (κ = 0.775 and 0.415, respectively) [16]. A possible explanation of the reduced reliability of strain in inferior and posterior-lateral circulation could be attributable to the problematic visualization of the posterior endocardium, which compromises image quality, or to the overlap between right and circumflex artery territories, that makes a precise separation of these territories not feasible.

The main limitation of this study is the relatively limited sample size of the included population. Moreover, in the current study, we enrolled all patients with suspected ischemia, including those with previous coronary artery disease which could have represented a potential bias. The analysis of coronary artery disease localization was based on a standardized perfusion model, without considering the overlap in posterior circulation and anatomic variation regarding the coronary artery dominance of each patients. Load dependence of GLS need to be accounted considering dynamic changes in afterload during DSE [19]. However, even though it has not been considered in the design of this study, the effect of load changes throughout the different stages of DSE can be expected to be negligible when RLS is considered. Finally, the exclusion of patients with negative DSE from the study didn’t allow the identification of true negative and false negative cases.

## Conclusions

AFI-based strain imaging analysis appears to be feasible during DSE even at the highest heart rate achieved at peak stress. Its use during DSE provides a slightly better agreement with coronary angiography results in presence of SCS of LAD, particularly in presence of resting wall motion abnormalities. Conversely, strain analysis correlates poorly with identification of SCS of both right and circumflex arteries, possibly due to scarce visualization of myocardial segments perfused by these two arteries and/or to perfusion territory overlap. Future multicenter study on larger population sample size are needed to test the usefulness of strain imaging during DSE.
